# Endocardial transvenous atrial pacing in lateral tunnel Fontan guided by electroanatomical mapping

**DOI:** 10.1016/j.ipej.2025.07.012

**Published:** 2025-07-26

**Authors:** Carlotta Sembenini, Alessandro Locatelli, Luca Tomasi, Giacomo Mugnai

**Affiliations:** Division of Cardiology, Cardio-Thoracic Department, University Hospital of Verona, Verona, Italy

**Keywords:** Fontan, Intracardiac tunnel, Transvenous lead, Endocardial pacing

## Abstract

The Fontan circulation is a palliative surgery used to treat cardiac abnormalities with only one well-developed ventricle. Although the surgical epicardial pacing is usually preferred, in a few cases the transvenous placement of a permanent atrial lead can be considered. The transvenous endocardial pacing is challenging because most of the right atrium is fibrotic and the lead positioning needs to be supported by a long sheath.

We report a case of a transvenous lead placement from a typical left pre-pectoral pocket guided by electro-anatomical mapping.

## 1 Introduction

**Fig. 1:**
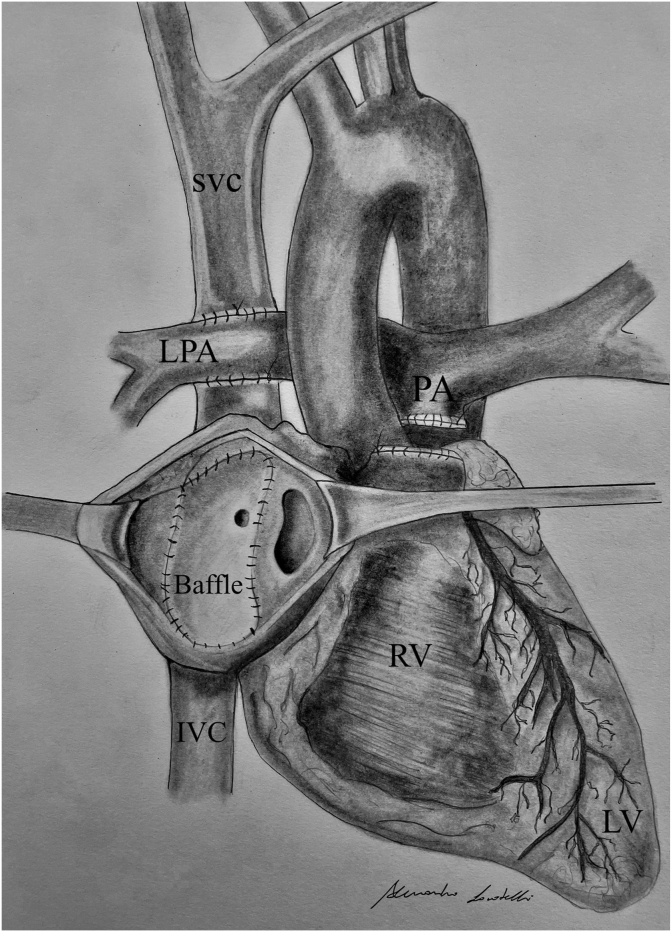
a manual drawing of the anatomy in the lateral tunnel Fontan surgery. SVC: superior vena cava. LPA: left pulmonary artery. PA: pulmonary artery. RV: right ventricle. IVC: inferior vena cava.

The Fontan-type operation is a palliative surgery for cardiac abnormalities with functionally univentricular hearts [1]. The two most common variants are represented by the extracardiac total cavopulmonary connection and the intracardiac lateral tunnel [2]. The latter consists of a direct connection between superior vena cava and pulmonary artery and the connection between inferior vena cava and pulmonary artery via an intracardiac tunnel where a baffle is created in order to address the inferior vena cava's blood to the pulmonary artery (Fig. 1). This surgical procedure has the disadvantage of multiple atrial suture lines; in addition, most of the atrium continues to be exposed to high venous pressure which increases the risk of sinus node dysfunction and bradyarrhythmias [3]. When feasible, epicardial pacing is usually preferred over the transvenous one because of increased risk of infection and thromboembolism of the latter [4,5]. The present report describes the placement of a transvenous lead from a typical left pre-pectoral pocket guided by electro-anatomical mapping.

## 2 Case presentation

A 30 year-old women presented to the emergency department for weakness and bradycardia. She had a history of congenital atresia of the tricuspid valve with hypoplasia of the right ventricle and normal connection of the main vessels. When she was 3 year-old she underwent Glenn surgery and then lateral tunnel Fontan surgery. The closure of an atrial septal defect using the Amplatzer system was performed in 2006; in 2009 she was operated for severe kyphoscoliosis. As she experienced two episodes of paroxysmal atrial fibrillation, amiodarone and oral anticoagulation therapy were started. The patient developed chronotropic incompetence manifested by recurrent junctional rhythm at 40 bpm and sinus node dysfunction. During the right heart catheterization, some posterior and lateral portions of the Fontan lateral tunnel could be captured by electrical stimulation. A significant reduction of pressure in pulmonary circulation and in the single ventricle was observed with right atrial pacing which resulted in a significant improvement of cardiac output. The case was discussed with a multidisciplinary team and, given the potential difficulty and risks with surgical epicardial pacing due to previous heart surgeries and severe kyphoscoliosis, implantation of an intravenous, intracavitary pacemaker was planned.

**Fig. 2:**
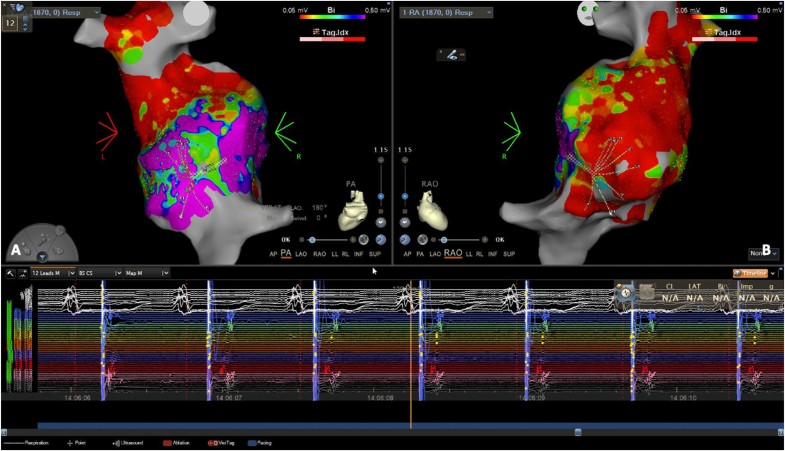
**A –** the postero-anterior view showed the Octaray voltage map of the posterior wall. **B –** the antero-posterior view showed the absence of electrical activity in the anterior wall of the right atrium. Below, the capture of atrial tissue was obtained by pacing from the Octaray mapping catheter placed in the posterior wall of the right atrium.

Under sedation and local anesthesia, after having punctured the right femoral vein, electro-anatomical bipolar map of the right atrium was reconstructed (CARTO, Bisoense&Webster, Irvine, California,USA) using a multipolar mapping catheter (Octaray, CARTO, Biosense&Webster, Irvine, California) (Fig. 2A–B). The posterior and lateral portion of the right atrium showed electrically active myocardial tissue assessed by the multipolar mapping catheter (Fig. 2A–B). After having accessed the left axillary vein under radioscopy guidance, the Selectra 3D 40-39 long sheath (Biotronik, Berlin, Germany) was used to achieve the target site in order to screw the active fixation lead (Solia S60, Biotronik, Berlin, Germany). The tip of the lead was visualized in the electro-anatomical map. The Octaray catheter was left in the target area to serve as a reference during fluoroscopic lead positioning. As expected, the atrial sensing was low (0.5 mV) and the pacing threshold was slightly elevated (2.6V@1ms) with normal impedance values.

**Fig. 3:**
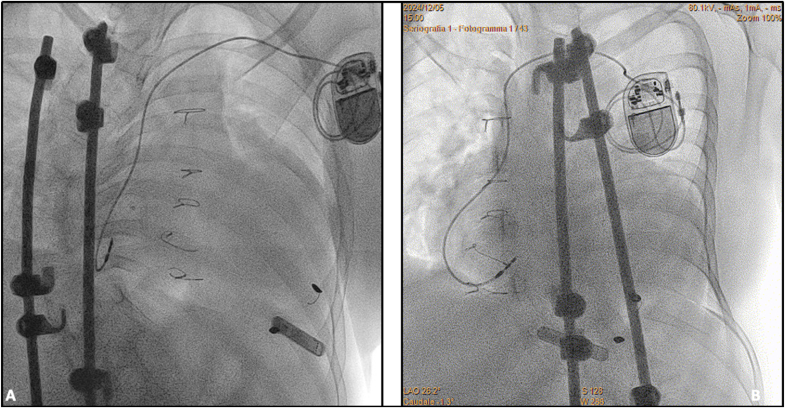
the X-ray image in right anterior oblique (RAO) view **(A)** and left anterior oblique (LAO) view showing the final position of the atrial lead.

The lead was connected to a single chamber pacemaker and programmed in the AAI mode. Prior to discharge, pacing threshold further improved to 1.2 V @ 1 ms with stable lead position, sensing and impedance values (Fig. 3).

## 3 Discussion

We report a case of successful implantation of an endocardial atrial lead for sinus node dysfunction in a patient with lateral tunnel Fontan. The transvenous procedure is usually challenging because the target site has to be reached and then the active fixation has to be supported by an appropriate long sheath. A few reports previously described the transvenous placement of an atrial lead in patients with lateral tunnel Fontan [[3], [4], [5]]. Hansky et al. [3] reported 5 out 7 cases of endocardial AAIR pacing after Fontan-type procedures using a de novo transvenous approach. The authors used a 4.1 French bipolar lumenless lead (SelectSecure, Medtronic, Minneapolis, MN, USA) supported by a deflectable sheath placed in the subpulmonary atrium. In the present case, the electro-anatomical mapping had a paramount role in order to evaluate where signals were still present in the atrium and to detect the best atrial portion for lead positioning. The voltage map showed that the best target area was in the posterior and lateral portion of the right atrium and for this reason the catheter was placed in this location. Although the pacing thresholds were slightly elevated during the implantation, the active fixation leads are commonly known to improve their pacing thresholds within a few hours.

During electro-anatomical mapping, atrial voltage of less than 0.05 mV is used to define scar and that more than 0.5 mV as healthy tissue. In the present case, the highest voltages were found in the posterior and lateral portions of the right atrium and the values of atrial sensing were slightly higher than 0.5 mV. However, as the right atrium was severely diseased and associated with a marked chronotropic incompetence, and the device was going to be programmed in order to constantly pace the atrium, an atrial sensing of 0.5 mV was deemed acceptable.

The understanding of atrial anatomy can be crucial in these patients and allows to test the health of the atrium (if any) and to precisely define the best target area where the atrial lead can be positioned with reasonable electrical parameters.

## Ethical statement

patient's consent has been taken for publication.

## Ethical statement

The patient has provided written informed consent for their clinical details and any accompanying images to be published in this journal. The patient was informed that all personal identifiers would be removed to protect their privacy, but that full anonymity cannot be absolutely guaranteed. The care provided to the patient was in line with our institutional ethical standards.

## Consent form for case reports

For a patient's consent to publication of information about them in a journal Title of article: Endocardial Transvenous Atrial Pacing in Lateral Tunnel Fontan Guided by Electroanatomical Mapping.

Medical practitioner or corresponding author: Giacomo Mugnai

I give my consent for this information about MYSELF relating to the subject matter above to appear in a journal article, or to be used for the purpose of a thesis or presentation.

I understand the following.

1. The Information will be published without my name/child's name/relatives name attached and every attempt will be made to ensure anonymity. I understand, however, that complete anonymity cannot be guaranteed. It is possible that somebody somewhere - perhaps, for example, somebody who looked after me/my child/relative, if I was in hospital, or a relative - may identify me.
2. The Information may be published in a journal which is read worldwide or an online journal. Journals are aimed mainly at health care professionals but may be seen by many non-doctors, including journalists.
3. The Information may be placed on a website.
4. I can withdraw my consent at any time before online publication, but once the Information has been committed to publication it will not be possible to withdraw the consent.

## Funding statement

the authors did not receive any funding relevant to the content of this paper.

## Declaration of competing interest

The authors declare that they have no known competing financial interests or personal relationships that could have appeared to influence the work reported in this paper.
